# Treatment outcome of oropharyngeal squamous cell carcinoma through propensity score analysis

**DOI:** 10.1016/j.bjorl.2023.101335

**Published:** 2023-09-23

**Authors:** Fabio Lau, Matheus Lisatchok, Jonas Belchior Tamanini, Fabio Portela Gazmenga, Daniel Naves Araújo Texeira, Eduardo Vieira Couto, Carlos Takahiro Chone

**Affiliations:** Universidade Estadual de Campinas (Unicamp), Departamento de Otorrinolaringologia e Cirurgia de Cabeça e Pescoço, São Paulo, SP, Brazil

**Keywords:** Squamous cell carcinoma, Oropharynx, Propensity score

## Abstract

According to an extensive database, the Objective is to compare surgical versus non-surgical treatment through Propensity Score (PS) for patients with Oropharyngeal Squamous Cell Carcinoma (OPSCC).

**Methods:**

We retrospectively evaluated epidemiological data from 8075 patients with OPSCC diagnosed between 2004 and 2014 and used PS matching to analyze possible prognostic factors for its outcomes with regression analyses.

**Results:**

Cox multiple regression analysis to study survival after PS matching shows that type of treatment was associated with death with a hazard ratio of 1.753 (*p* < 0.05) of non-surgical treatment. However, it was not associated with recurrence (*p* > 0.05). In the surgical treatment group, overall survival was 79.9% at one year, 36.4% at five years, and 20.5% at ten years. Disease-free survival was 90.1%, 64.8%, and 56.0% at 1, 5, and 10-years, respectively. In the non-surgical treatment group, overall survival was 60.6% at one year, 21.8% at five years, and 12.7% at ten years. Disease-free survival was 90.8%, 67.2%, and 57.8% at 1, 5, and 10-years, respectively.

**Conclusion:**

Patients in the surgical treatment group had better outcomes related to survival. Recurrence is associated with the survival of OPSCC cancer. Recurrence-free survival is similar to both treatments.

**Level of evidence:**

2C.

## Highlights

• Type of treatment was associated with death, but not with recurrence.
• In the surgical treatment group, overall survival was 36.4% at five years.
• In the non-surgical treatment group, overall survival was 21.8% at five years.
• In the surgical treatment group, disease-free survival was 64.8% at five years.
• In the non-surgical treatment group, disease-free survival 67.2% at five years.

## Introduction

According to International Agency for Research on Cancer (IARC), there were 98.412 new cases of oropharyngeal cancer worldwide and 5308 in Brazil in 2020. This cancer was responsible for 48.100 deaths worldwide that year, of which 3248 were Brazilian patients. The incidence of this cancer is increasing, with an estimated 145.000 new cases and mortality of 73.400 patients in 2040.^1^ The geographic variation in the global incidence of Oropharyngeal Squamous Cell Carcinoma (OPSCC) is attributed to cultural differences involving risk factors such as alcohol and tobacco consumption, prevalence of Human Papillomavirus (HPV) infection.^2^ However, even with a decline in tobacco use,^3^ there has been an increase in the incidence of OPSCC, especially in HPV- positive patients, unlike other head and neck neoplasms.^4^

The oropharynx is a difficult-to-access anatomic area, and a suspicious lesion may go unnoticed until the advanced stages. Moreover, early staged lesions can still be missed, especially because they are frequently asymptomatic in the majority. Studies report diagnosis in stages III or IV in 75%–81% of cases.^5^, ^6^

The optimal treatment for patients with OPSCC remains controversial. Currently, there are several treatment options, each with different limitations, sequelae, and outcomes. Patients with early-stage OPSCC have similar outcomes to Radiotherapy (RT) or surgery.^7^ However, most patients have advanced-stage disease at diagnosis in some populations,^5^, ^6^ and there is no consensus on the best management of these cases.^8^, ^9^, ^10^

Until recently, there were no prospective Randomized Controlled Trials (RCT) comparing a primary surgical versus non-surgical approach to inform treatment decisions.^11^, ^12^ Most studies were heterogeneous and retrospective.^11^ However, for data from observational studies, the statistical analyses are often biased due to treatment-selection factors. Propensity Score (PS) based methods estimate the survival function by adjusting for the disparity of PS between treatment groups. This method has been shown as a possible alternative for prospective RCT.^13^ The present study aimed to compare the surgical versus non-surgical treatment of OPSCC in large databases through PS evaluating prognostic factors related to disease-free and overall survival.

## Methods

We analyzed data from the São Paulo Cancer Center Foundation (*Fundação Oncocentro de São Paulo*, or FOSP for short, in Portuguese) database relative to patients with OPSCC diagnosed between 2004 and 2014 in Sao Paulo.

The database is available on FOSP official website at http://www.fosp.saude.sp.gov.br/publicacoes/rhc. The data are in the public domain and not nominal. By the policy of the Brazilian National Research Ethics Committee (CEP/PRP No. 068/202), studies using publicly available datasets are exempt from institutional research ethics committee approval as they do not involve human subjects.

We selected the anatomic sites for oropharyngeal cancer development based on topographic diagnosis according to the International Classification of Diseases for Oncology (ICD-O) second edition until the end of 2005 and ICD-O third edition from 2006 onwards. Initially, all patients listed in the database with an ICD-O code corresponding to a neoplasm in an anatomic site related to the oropharynx were eligible for inclusion. Subsequently, patients without a diagnosis of OPSCC were excluded. Age, sex, clinical stage at diagnosis, type of treatment, and overall recurrence were assessed with death as an outcome.

Statistical analysis was performed using the SAS System for Windows (Statistical Analysis System), version 9.4. For descriptive statistics, categorical variables were expressed as numbers (n) and percentages (%), and numerical variables as mean (SD) or median (range). To compare proportions, the chi-square test was used. The Mann-Whitney test was applied to compare continuous measurements between 2 groups. The PS was used to control the potential confounding factors in the association between the characteristics and the outcome. Surgical and non-surgical treatment cases were considered to construct the PS and patients without information on the variables of interest were excluded (Other treatment combinations and No treatment performed). Univariate and multivariate Cox regression analyses were performed to assess factors associated with the outcomes. In our study, the PS technique was applied, which consisted of modeling the treatment about the age, sex, clinical stage at diagnosis, type of treatment, and the time between the beginning of treatment and recurrence on a logistic regression model.

The quality of the estimated propensity score in a sample is assessed by comparing the distributions between the two treatment groups. If the propensity score distribution is different in the two treatment groups, it may compromise the analysis. Thus, it was decided to match the treatments in a ratio of 1 to 1, and the sample was reduced to 3818 which 1909 were treated with surgery, and 1909 were treated with a non-surgical approach. In the matched data obtained by the PS, the regression Cox analysis was repeated only with treatment as an independent variable, and the survival curves were estimated. The level of significance was set at 5% for all comments.

## Results

The logistic regression analysis was used to balance and calculate the PS modeling surgical versus non-surgical treatment. The variables considered were gender, age, stage, and time between diagnosis and the beginning of treatment. The Cox multiple regression outcomes to death and recurrence using PS as covariate shows that treatment type and recurrence were associated with death with a hazard ratio of 1,594 and 1,122, respectively (*p* < 0.05). However, treatment was not associated with recurrence after PS (*p* > 0.005).

The descriptive analysis of the variables selected after PS and matching are shown in Table 1, Table 2. In the non-surgical treatment group, the mean age was 56.6 (SD = 10.18); the mean time between diagnosis and treatment was 64.91 (SD = 69.22); men (85.4%) were more affected than women (14.6%); the tonsils and base of the tongue represent 45.9% while other anatomical sites representing 54.1% of the cases; 22.7% died and 17% had a recurrence. In the surgical treatment group, the mean and median age was 57.24 (SD = 9.71); the mean time between diagnosis and treatment was 60.11 (SD = 85.85); men (88.3%) were more affected than women (11.7%); the tonsils and base of the tongue represent 44.6% while other anatomical sites representing 55.4% of the cases; 32.5% died and 25.4% had a recurrence.

**Table 1:** Descriptive analysis and comparisons of selected variables for propensity score between treatments.

Variable	Non-surgical treatment (n = 4640)	Surgical treatment (n = 2200)	*p*-value
Age			
Mean ± SD (n)	57.95 ± 10.10 (n = 4640)	57.28 ± 9.82 (n = 2200)	0.284^a^
Median (min‒max)	57.00 (20.00–99.00)	57.00 (9.00–92.00)	<0.001^a^
Sex			
Male	4183 (90.2%)	1905 (86.6%)	<0.0001^b^
Female	457 (9.8%)	295 (13.4%)	<0.0001^b^
Anatomic Site			
Base of tongue/Tonsil	1966 (42.4%)	974 (44.2%)	0.1377^b^
Other sites	2674 (57.6%)	1226 (55.7%)	0.1377^b^
Stage			
Early	342 (7.5%)	567 (26.5%)	<0.0001^b^
Advanced	4190 (92.5%)	1575 (73.5%)	<0.0001^b^

a Based on Mann–Whitney test.

b Based on the Chi-Square test.

**Table 2:** Descriptive analysis of the selected variables after propensity score and matching between treatments.

Variable	Non-surgical treatment (n = 1909)	Surgical treatment (n = 1909)
Age		
Mean ± SD (n)	56.60 ± 10.18 (n = 1909)	57.24 ± 9.71 (n = 1909)
Median (min‒max)	56.00 (20.00–99.00)	57.00 (9.00–92.00)
Sex		
Male	1630 (85.4%)	1685 (88.2%)
Female	279 (14.6%)	224 (11.7%)
Anatomic Site		
Base of Tongue/Tonsil	876 (45.9%)	851 (44.6%)
Other sites	1033 (54.1%)	1058 (55.4%)
Stage		
Early	335 (17.5%)	340 (17.8%)
Advanced	1574 (82.5%)	1569 (82.2%)
Death		
No	335 (17.5%)	340 (17.8%)
Yes	1574 (82.5%)	1569 (82.2%)
Recurrence		
Yes	324 (17.0%)	484 (25.4%)
No	1585 (83.0%)	1425 (74.6%)

**Figure 1:**
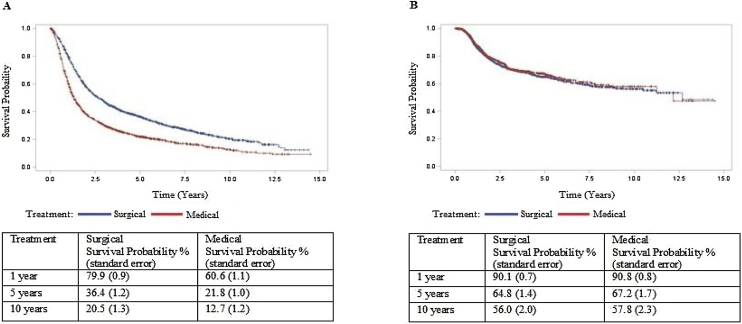
Survival curves comparing Surgical × Non-surgical treatment after propensity score and matching estimated by Cox regression. (A) Overall survival time curve. (B) Disease free survival time curve.

Cox multiple regression analysis to study survival after PS matching shows that treatment and recurrence were associated with death with a hazard ratio of 1,753 and 1,599, respectively (*p* < 0.05). However, treatment was not associated with a recurrence rate after PS matching (Table 3). Cox regression was repeated only with the treatment type variable and estimated survival curves (Fig. 1). In the surgical treatment group, overall survival was 79.9% at one year, 36.4% at five years, and 20.5% at ten years. Disease-free survival was 90.1%, 64.8%, and 56.0% at 1, 5, and 10-years, respectively. In the non-surgical treatment group, overall survival was 60.6% at one year, 21.8% at five years, and 12.7% at ten years. Disease-free survival was 90.8%, 67.2%, and 57.8% at 1, 5, and 10 years, respectively.

**Table 3:** Cox multiple regression analysis to study survival and recurrence after propensity score matching.

Variable	Category	*p*-value	HR	95% CI
Death				
Treatment	Non-surgical × Surgical	<0.0001	1.753	1.583‒1.943
Recurrence	Yes × No	<0.0001	1.599	1.332‒1.919
Recurrence				
Treatment	Surgical × Non-surgical	0.5885	1.055	0.870‒1.279

HR, Hazard Ratio; 95%CI, Confidence Interval for the ratio, matching treatment for propensity score.

## Discussion

The optimal treatment for patients with OPSCC remains up to the ideal evidence practice scenario without multicentric randomized prospective trials.^11^, ^12^ Surgical or non-surgical treatment has specific benefits, limitations, sequelae, and outcomes.

Althoug the intensity-modulated radiation has reduced some adverse effects of this therapy, the patient with OPSCC may still have xerostomia, mucositis, dysphagia and feeding tubes dependency after 6-months from RT.^14^ Surgical treatment could allow the identification of positive margins, extracapsular spread, perineural invasion, and adequate histopathologic staging of the neoplasm that guide the choice of patients who would benefit from adjuvant therapy but with a cost of swallowing morbidities even with best reconstructive techniques.

Some surgical techniques have been developed to reduce patient morbidity while maintaining satisfactory oncological outcomes for early cancers. Transoral approaches have gained favor compared to open surgical procedures in treating OPSCC due to shorter length of stay in the hospital, lower risk of free flap reconstruction, and shorter time to decannulation.^15^ However, transoral surgery as access can be limited in patients with severe trismus, obesity, limited neck extension.^15^, ^16^ In a multicenter study of patients with advanced OPSCC from the United States, Haughey et al., showed three-year overall survival, disease-specific survival, and disease-free survival were 86%, 88%, and 82%, respectively.^17^ But concerns are discussed considering the lack of multicenter randomized trials comparing open surgical, robotic, and non-surgical arms.

Primary RT and surgery are equally effective in patients with early-stage OPSCC. Morisod et al.^18^ 70's meta-analysis showed that 5-year disease-specific survival/overall survival was 90.4% in the RT group versus 89.6% in the surgical group, suggesting equivalent efficacy of both treatments. For the advanced stage, recent retrospective studies reported improved survival in patients submitted to primary surgery with adjuvant therapy compared to primary Chemoradiotherapy (CRT). Kamran et al.^19^ compared surgery with or without RT ± chemotherapy versus primary CRT treatment for locally advanced oropharyngeal cancer and found that 3-year survival was 85.4% and 72.6% (*p* < 0.0001), respectively. Zenga et al.^20^ also showed that primary surgical treatment might be associated with improved outcomes in patients with T4 OPSCC compared to non-surgical treatments. The systematic review of Yeh et al.^21^ comparing surgery versus RT for the management of OPSCC reports that surgery has similar survival outcomes. Still, functional outcomes may be superior when compared to non-surgical therapy. However, the authors highlighted that differences in patient populations could explain these findings. Surgery studies tended to have fewer advanced tumors and cervical metastasis than non-surgical series. Moreover, surgery patients are usually carefully selected because their tumors were thoroughly evaluated for resectability with clear margins. Finally, the authors also suggested that some studies may have publication bias since some institutions might refrain from publishing their outcomes if not according to the published data in the literature.^21^

Thus, there is a paucity of prospective RCT comparing a primary surgical versus primary RT approach for T1/T2 cancers, and T3/T4 ones.^11^ ORATOR^22^ is the only RCT published that compared patients with T1‒T2 N0-N2 (LN ≤ 4 cm) treated with primary RT versus those treated with transoral surgery and showed that RT arm had superior swallowing-related QOL scores one year after treatment. However, the difference became less pronounced at a long-term follow-up.^22^ Despite the efforts to present the first RCT to compare transoral surgery versus primary RT for the treatment of OPSCC, there are some limitations that we have to consider. The number of patients is 34; only ten were treated with surgery alone, while 16 received dual modality therapy, and eight received triple modality therapy. Moreover, the study included only patients with T1‒T2 N0‒N2, which is not the reality of some populations.^5^, ^6^ According to our database, 86.3% of patients of OPSCC were diagnosed at an advanced stage. We must be critical in applying the ORATOR conclusions to lower-income countries, especially when approaching patients from the public health system.

For observational studies, the statistical analyses about survival and disease survival are often biased due to treatment-selection factors. Many considerations influence the selection of one therapy over another, like tumor staging, site of tumor presentation, lymphatic metastases, institution expertise, and patient preference. In many settings, more than one therapeutic approach is used, usually a multimodality treatment. Advanced cancer is often treated with a multimodal approach. As the researcher has no control over allocating individuals to groups, the probability distributions of the covariates may be different, and the groups are not fully comparable in our database. The PS is the probability that a patient would receive the treatment of interest based on confounders such as patient characteristics, treating clinician, and clinical environment. This method reduces the bias in estimating treatment effects and the likelihood of confounding when analyzing nonrandomized, observational data. Cox multiple regression analysis to study survival after PS matching shows that patients undergoing non-surgical treatment and with recurrence were more likely to die (HR: 1,753 and 1.599, respectively). However, our study could not observe the type of treatment as a recurrence predictor after PS matching (*p* > 0.05). This could be due to the incompleteness of the record of recurrences regarding the retrospective nature of our research, selection bias considering non-surgical treatment for patients unfit in medical conditions for surgery, or they die due to aggressive non-surgical treatment in poor health conditions. These medical conditions are not described in the database records and could not be balanced between groups.

HPV status could not be used as a prognostic factor as the database did not record this information. But HPV evaluation was only recently implemented and the dataset back to 2004. Complete HPV evaluation is not available with hybridization-in-situ or DNA PCR but solely with P16 immunohistochemistry with a limited resources in public health insurance. Thus, this data could be a source of bias and represents a limitation of our study. However, the positive status HPV in Brazilian population seems to be lower than United States and Europe.^23^, ^24^ Although patients with HPV-positive OPSCC have more lymphatic metastases,^25^ have better overall survival and disease-free survival rates than those with HPV-negative OPSCC, regardless of the type of treatment.^26^

There are inherent limitations in a population-based study, such as the need for more information on comorbidities, type of surgery performed, and reason for non- surgical treatment. The choice of nonsurgical treatment because of complete tumor resectability or impaired functional status may determine selection bias since patients within this profile are more likely to have adverse outcomes, regardless of treatment type. Additionally, we could not collect data on functional outcomes and quality of life, which are relevant factors in the comparison of treatment modalities since HPV-positive OPSCC patients are typically younger and healthier with improved survival compared to patients with HPV-negative, smoking-related head and neck squamous cell carcinoma.^26^, ^27^

Although it is a retrospective observational study, we used the PS to estimate survival adjusted for the disparity between treatment groups and reduce the selection bias. This method is an excellent alternative to prospective randomized studies in their absence. This is the first study to use PS to compare surgical and non-surgical treatment in patients with OPSCC in a matched groups by age, gender, stage, and site. This method showed no significant difference in disease-free survival between patients receiving surgical and non-surgical treatment, suggesting similar complete remission rates with both approaches. But those who receive surgical treatment live far well, offering better outcomes in surgical arms.

## Conclusion

Patients in the surgical treatment group had better outcomes related to survival. Recurrence is associated with the survival of OPSCC cancer. Recurrence-free survival is similar to both treatments.

## Authors contributions

Matheus Lisatchok: Contributed to drafting the manuscript.

Jonas Belchior Tamanini: Contributed to drafting the manuscript.

Fabio Portela Gazmenga: Contributed to drafting the manuscript.

Daniel Naves Araújo Texeira: Contributed to drafting and revising the manuscript critically for important intellectual content.

Eduardo Vieira Couto: Contributed to drafting and revising the manuscript critically for important intellectual content.

Carlos Takahiro Chone: Contributed to the conception and design; Acquisition, analysis, and interpretation of data.

## Funding

None to declare.

## Conflicts of interest

The authors declare no conflicts of interest.
